# Generalized spike–waves in idiopathic generalized epilepsies: Does their frequency matter?

**DOI:** 10.1002/brb3.70023

**Published:** 2024-10-04

**Authors:** Ali A. Asadi‐Pooya, Mohsen Farazdaghi

**Affiliations:** ^1^ Epilepsy Research Center Shiraz University of Medical Sciences Shiraz Iran; ^2^ Jefferson Comprehensive Epilepsy Center Department of Neurology Thomas Jefferson University Philadelphia Pennsylvania USA

**Keywords:** EEG, epilepsy, outcome, seizure

## Abstract

**Objectives:**

We hypothesized that the frequency (in Hertz) of generalized spike–waves (GSWs) in patients with idiopathic generalized epilepsy (IGE) has associations with the syndromic diagnosis as well as with the prognosis of patients (their response to medical treatment).

**Methods:**

This was a retrospective study of a prospectively developed database. All patients with a diagnosis of IGE were studied at the epilepsy center at Shiraz University of Medical Sciences, Shiraz, Iran, from 2008 until 2022. Patients were classified into four IGE syndromes: childhood absence epilepsy; juvenile absence epilepsy; juvenile myoclonic epilepsy; and generalized tonic–clonic seizures alone.

**Results:**

Five hundred and eighty‐three patients were studied. GSWs were commonly observed in all four syndromes of IGE. Frequency of GSW (in Hertz) did not have a significant association with the syndromic diagnosis of the patients (*p* = .179). The presence of GSW did not have a significant association with the seizure outcome (becoming seizure free or not) of the patients (*p* = .416). Frequency of GSW did not have a significant association with the seizure outcome of the patients either (*p* = .574).

**Conclusion:**

GSWs are the hallmark electroencephalographic footprints of idiopathic generalized epilepsies; however, neither their presence nor their frequency has practical associations with the syndromic diagnosis of IGEs or their outcome (response to treatment).

## Graphical abstract

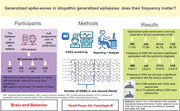

## 1 INTRODUCTION

Idiopathic generalized epilepsies (IGEs) are genetic epilepsy syndromes diagnosed by certain clinical and electroencephalographic (EEG) characteristics proposed by the International League Against Epilepsy (ILAE) (Hirsch et al., 2022). They constitute about 20% of all patients with epilepsy (PWE) (Asadi‐Pooya, Emami, & Sperling, 2013; Hirsch et al., 2022). There are four IGE syndromes recognized by the ILAE: childhood absence epilepsy (CAE); juvenile absence epilepsy (JAE); juvenile myoclonic epilepsy (JME); and generalized tonic–clonic seizures alone (GTCA) (Hirsch et al., 2022).

Electroencephalography in patients with IGE may show generalized spike–waves (GSWs) (≥2.5 Hz) and/or polyspike–wave discharges (Asadi‐Pooya, Emami, & Sperling, 2013; Cerulli Irelli et al., 2022; Hirsch et al., 2022). In other words, EEG may include a wide range of frequencies (the number of waves that pass by each second) for GSW in different patients. This wide range may bring about two questions: (1) What does determine the frequency of GSW? (2) Does the frequency of GSW matter in clinical practice (does it have any diagnostic or prognostic implications)?

The aim of the current clinical study was to provide an answer to the latter question. We hypothesized that the frequency of GSW has associations with the syndromic diagnosis as well as with the prognosis of patients (their response to medical treatment).

## 2 METHODS

### 2.1 Participants

This was a retrospective study based on a large database of PWE that was built prospectively over 14 years. The seizure types and epilepsy syndromes were diagnosed by the senior epileptologist at our center. All the patients had an electroencephalography performed at our epilepsy center, but an abnormal EEG result was not needed to make a final diagnosis of an IGE syndrome (when a detailed clinical history was compatible with a diagnosis of an IGE syndrome). All patients with a diagnosis of IGE were studied at the epilepsy center at Shiraz University of Medical Sciences, Shiraz, Iran, from 2008 to 2022. Patients were classified into IGE syndromes (i.e., CAE, JAE, JME, and GTCA) according to the ILAE definitions (Hirsch et al., 2022). Patients with comorbid functional seizure and those with missing data were excluded.

### 2.2 Data collection

Sex, age, age at seizure onset, seizure type(s), seizure frequency, EEG findings, the syndromic diagnosis, and treatment plan were routinely registered for each patient at the first visit and during the follow‐up visits. We investigated whether the patients were seizure‐free for 1 year (12 months) after their initial visit. For each patient, a 2‐h video‐EEG monitoring was done. Each EEG at least consisted 1 hour of sleep. Activation methods, including sleep deprivation, hyperventilation, and photic stimulation, were used for all patients. The EEG was done before starting or switching the antiseizure medication(s). We visually measured and recorded the frequency (i.e., Hertz [Hz] during the first second of a burst or a single‐GSW complex when there were no bursts) of GSW that happened in wakefulness (unless they happened only during sleep).

### 2.3 Statistical analyses

The IBM SPSS Statistics (version 25.0) was used for the statistical analyses. Values were presented as mean ± standard deviation for continuous variables and as number (percent) of subjects for categorical variables. Pearson chi‐square test, Fisher's exact test, one‐way ANOVA (analysis of variance), and independent *t*‐test were used for statistical analyses. A *p* value (2‐sided) less than  .05 was considered significant.

### 2.4 Standard protocol approvals, registrations, and patient consents

The Shiraz University of Medical Sciences Institutional Review Board approved this study (IR.SUMS.REC.1401.412). Informed consent was obtained from all the participants (to include their data in the database).

## 3 RESULTS

### 3.1 Characteristics of the patients

During the study period, 4304 patients were registered at our epilepsy center. Five hundred and eighty‐three patients had IGE and fulfilled the inclusion criteria of the study. The demographic and clinical characteristics of the patients are summarized in Table 1. Two hundred and ninety‐two patients (50.1%) had JME, 118 people (20.2%) had JAE, 98 individuals (16.8%) had GTCA, and 75 patients (12.9%) had CAE.

**TABLE 1:** The demographic and clinical characteristics of the studied patients with idiopathic generalized epilepsy.

Variables	Numbers (total = 583) and percents
Sex (female: male) (%)	352 (60.4%): 231 (39.6%)
Age at seizure onset, years (±SD*)	13.2 ± 6.3 (min. 1.2; max. 74; median 21)
Age at diagnosis, years (±SD)	21.8 ± 9.7 (min. 0.1; max. 54; median 13)
Normal EEG† (%)	74 (12.7%)
Generalized spike–waves (%)	451 (77.4%)
Frequency of generalized spike–waves, Hz (±SD)	3.5 ± 0.6 (min. 2.5; max. 6; median 3.5)
Frequency of generalized spike–waves, Hz (±SD) in syndromes (JME, JAE, GTCA, and CAE)	3.6 ± 0.7, 3.5 ± 0.5, 3.5 ± 0.6, 3.4 ± 0.5
Polyspikes (%)	309 (53.0%)
Photosensitivity during EEG (%)	53 (9.1%)

Abbreviations: CAE, childhood absence epilepsy; EEG, electroencephalographic; GTCA, generalized tonic–clonic seizures alone; JAE, juvenile absence epilepsy; JME, juvenile myoclonic epilepsy.

*Standard deviation.

†Electroencephalography.

One hundred and sixty‐four patients (28.1%) were newly diagnosed at our center, and 419 individuals (71.9%) were referred to us by other physicians (often due to uncontrolled seizures). Five hundred and five patients (86.8%) received monotherapy with ASMs, 72 individuals (12.3%) were prescribed polytherapy with ASMs, and 6 persons (1%) decided not to take any ASMs (first seizure [4 patients] or myoclonic seizures only [2 patients]). At the time of referral, some patients were taking different ASMs (e.g., carbamazepine, sodium valproate, lamotrigine, and topiramate). The distribution of data was normal.

The frequency of GSWs did not differ between men and women (*p* = .424). Moreover, taking sodium valproate was not associated with any difference in the GSW frequency (*p* = .553).

### 3.2 Does the frequency of GSW have association with the syndromic diagnosis in IGE?

GSWs were commonly observed in all four syndromes of IGE. However, the presence of GSW had a significant statistical association with the syndromic diagnosis of the patients: GSWs were seen in 89.3% (*N* = 67) of patients with CAE, 84.7% (*N* = 83) of those with GTCA, 77.9% (*N* = 92) of patients with JAE, and in 71.6% (*N* = 209) of individuals with JME (*p* = .002; degree of freedom = 3; Pearson chi‐square test).

Frequency of GSW did not have a significant association with the syndromic diagnosis of the patients (*p* = .179; one‐way ANOVA). In the two‐by‐two comparison of CAE (a syndrome with a higher presence of GSW) and JME (a syndrome with a lower presence of GSW), the frequency of GSW did not have a significant association with the syndromic diagnosis of the patients either (GSW frequency of 3.6 ± 0.7 Hz in JME and 3.4 ± 0.5 Hz in CAE; *p* = .069; *t*‐test).

### 3.3 Does the frequency of GSW have association with the outcome in IGE?

Three hundred and fifty‐eight patients had at least 12 months of follow‐up at our center and were included in the outcome analysis. One hundred and three patients (28.8%) had new‐onset epilepsy, and 255 individuals (71.2%) were referred to us by other physicians (often due to uncontrolled seizures). Three hundred and eight patients (86.0%) were on monotherapy with ASMs, 49 individuals (13.7%) were prescribed polytherapy with ASMs, and one person (0.3%) decided not to take any ASMs (with a single seizure). One hundred and thirty‐four patients (37.4%) were free of all seizure types, and 224 people (62.6%) were not seizure‐free. The presence of GSW did not have a significant association with the seizure outcome of the patients: A total of 104 out of 286 patients with GSW and 30 out of 72 individuals without GSW were seizure‐free at 12 months of their follow‐up (*p* = .416; degree of freedom = 1; Fisher's exact test). Frequency of GSW did not have a significant association with the seizure outcome of the patients either (*p* = .574; *t*‐test).

In a subanalysis with patients who had JME and at least 12 months of follow‐up at our center (*N* = 180), similar results were obtained: The presence of GSW did not have a significant association with the seizure outcome (*p* = .342; degree of freedom = 1; Fisher's exact test), and frequency of GSW did not have a significant association with the seizure outcome either (*p* = .725; *t*‐test). For other syndromes, the numbers were small for any syndrome‐specific statistical analysis.

## 4 DISCUSSION

The major role players in generating typical spike–wave discharges (≥2.5 Hz) are selective bidirectional thalamo‐cortical communications (i.e., circuits and networks). Spike–waves can be deemed a final common brain output, resulting from an inherent tendency of circuits and networks to oscillate between excitatory and inhibitory activity; this may be triggered by a variety of different pathophysiological etiologies (Blumenfeld, 2005; McCafferty et al., 2018). Therefore, it is expected to see a variety of frequencies of GSW in different patients with IGE; a variety of underlying etiologies and pathophysiological processes (e.g., different genetic problems) create a variety of thalamo‐cortical circuit and network problems that manifest as GSW (as a final output), but with different frequencies. The exact mechanisms underlying GSW characteristics (e.g., their frequency, their abundance, and their duration) should be clarified in future studies.

**FIGURE 1:**
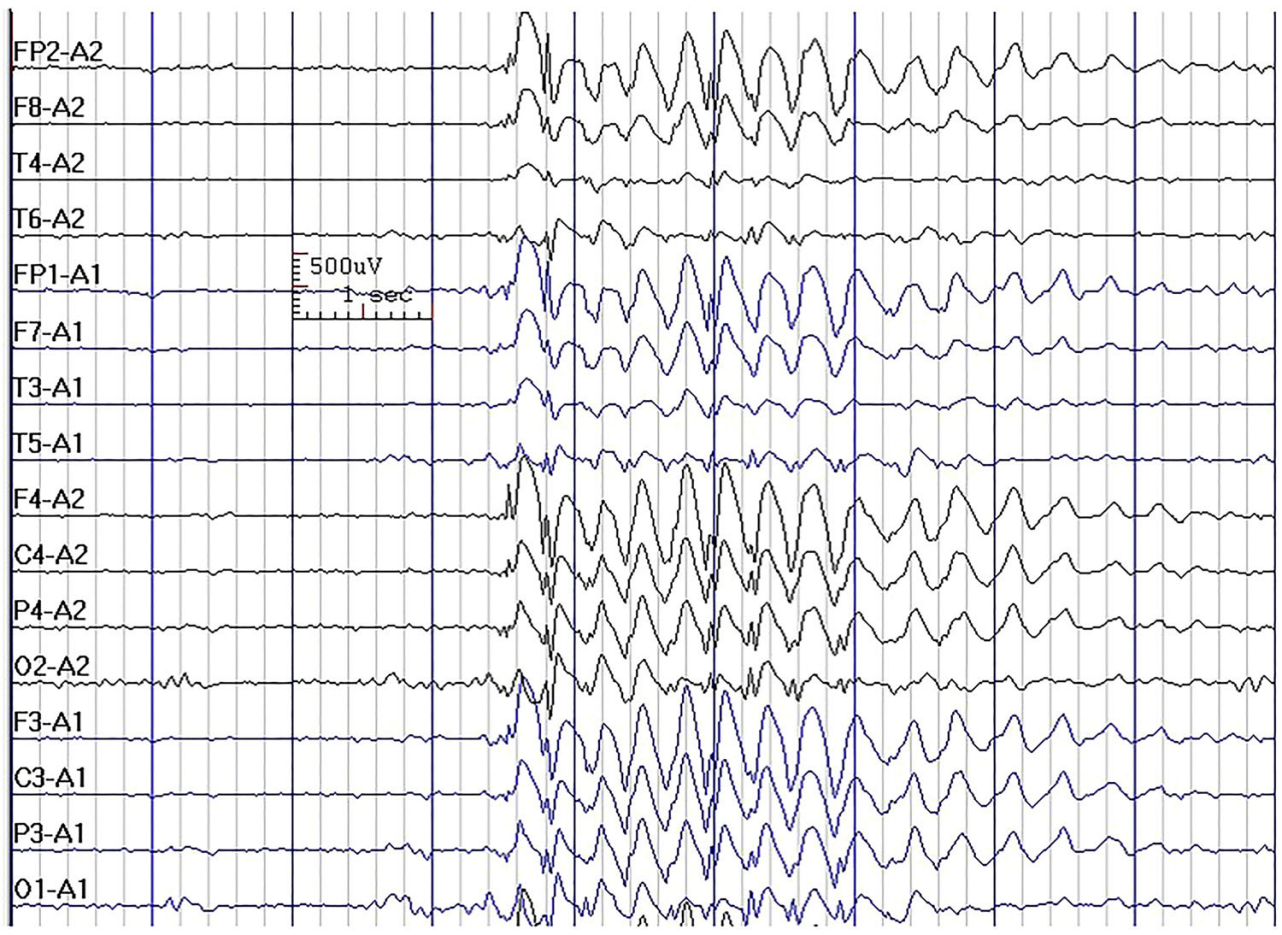
Generalized 3.5 Hz spike–waves in a 16‐year‐old girl with juvenile absence epilepsy.

In the current study, we observed that GSWs (Figure 1) were commonly observed in all four syndromes of IGE, and although the presence of GSW had a significant statistical association with the syndromic diagnosis of patients, it was not discriminatory between the syndromes (e.g., 89.3% in CAE vs. 71.6% in JME) and, therefore, does not have any practical diagnostic implications. Furthermore, we observed that the frequency of GSW did not have a significant association with the syndromic diagnosis of the patients. In a previous study, we reported that interictal EEG cannot differentiate between the seizure types in IGEs (Asadi‐Pooya & Emami, 2012b). Moreover, it was previously shown that the morphology, amplitude, duration, frequency, occurrence, or activation of the GSWs did not differ between the classic adolescent‐onset and the adult‐onset IGEs (Yenjun et al., 2003).

Another study reported that polyspike and waves do not predict the presence of generalized tonic–clonic seizures in CAE (Vierck et al., 2010). We can conclude that GSW (variable frequencies) and polyspikes, alone or in combination, could be observed in various seizure types and syndromes of IGE. Although EEG is a very helpful ancillary test to differentiate IGEs from other epilepsy syndromes (e.g., focal epilepsies), the key element in making a correct syndromic diagnosis is a detailed clinical history (Asadi‐Pooya & Emami, 2012a; Asadi‐Pooya, Emami, Ashjazadeh, et al., 2013).

A previous study reported that the density and duration of epileptiform discharges can help differentiate among IGE syndromes (Seneviratne, Hepworth, et al., 2017). In a study of 105 patients, the authors reported that the density of epileptiform discharges and the paroxysm durations were the highest in JAE, followed by JME, CAE, and GTCA (no clear cutoff discriminatory value was provided). They reported that “pure” GSW paroxysms and fragments were observed in all four IGE syndromes (Seneviratne, Hepworth, et al., 2017).

Again, although their findings were statistically significant, those observations could not have significant practical implications in daily clinical practice; there was no pathognomonic or characteristic EEG signature for any IGE syndrome. These kinds of statistically significant EEG differences between IGE syndromes (without clear practical and clinical implications) have also been reported by other groups (Sadleir et al., 2009).

In the current study, we also observed that neither the presence of GSW nor their frequency had significant associations with the seizure outcome of the patients with IGE. This intriguing observation should be further explored in future studies. Having said the above, EEG may help with the prognostication in PWE.

In 1 previous study of 105 patients with IGE, the authors reported that higher densities and longer durations of epileptiform discharges on EEG were associated with a shorter duration of seizure freedom (Seneviratne, Boston, et al., 2017). Another study showed that prolonged epileptic discharges predict seizure recurrence and ASM failure in JME (Seneviratne, Boston, et al., 2017). One study of prognostic factors for CAE (*n* = 53) and JAE (*n* = 27) showed that the presence of polyspikes during sleep was associated with poor prognosis in CAE; no statistical correlation was found for JAE (Bartolomei et al., 1997).

In a recent study of 232 patients with IGE, increased GSW in sleep and the presence of generalized polyspike trains were associated with drug‐resistance (Kamitaki et al., 2022). Therefore, although interictal EEG can potentially be used as a biomarker of prognosis in IGE syndromes, the mere presence or frequency of GSW does not provide a value here.

Our study has significant limitations. This was a retrospective study. In addition, most patients (71.9%) were referred to us (often due to uncontrolled seizures for years). This may explain the observed outcome at 12 months of follow‐up (only 37.4% were seizure‐free of all seizure types). Although the proportion of patients who were not seizure free was quite high, the number of patients, who were on monotherapy, was also quite high (86%); this is because we investigated their outcome during the first 12 months since their initial visit. Furthermore, the EEG was inspected visually only. In the current study, we visually measured and recorded the initial frequency of GSW that happened in wakefulness (unless they happened only during sleep); however, we did not record that in how many patients the EEG abnormality happened only during sleep. Finally, although the EEG was done before starting or switching the ASM, the possible effect of the previous ASM(s) was not evaluated.

## 5 CONCLUSION

GSWs are the hallmark EEG footprints of idiopathic generalized epilepsies; however, neither their presence nor their frequency has practical associations with the syndromic diagnosis of IGEs or their outcome (response to treatment). A detailed clinical history is the key to make a correct syndromic diagnosis, and this diagnosis establishes the foundation for the treating physician to decide on an appropriate treatment strategy as well as to explain the prognosis for the patient and their caregivers.

## AUTHOR CONTRIBUTIONS

**Ali A. Asadi‐Pooya**: Study design; data collection; statistical analysis; and manuscript preparation. **Mohsen Farazdaghi**: Data collection and manuscript preparation.

## CONFLICT OF INTEREST STATEMENT

Ali A. Asadi‐Pooya: Honoraria from Cobel Daruo, Ronak, and RaymandRad; Royalty: Oxford University Press (book publication); grant from the National Institute for Medical Research Development. Others: none.

### PEER REVIEW

The peer review history for this article is available at https://publons.com/publon/10.1002/brb3.70023

## INFORMED CONSENT

Informed consent for participation and publication of the data were obtained from all patients.

## Data Availability

The data would be shared as per the regulations of Shiraz University of Medical Sciences.
